# Resource trading strategies with risk selection in collaborative training market

**DOI:** 10.1371/journal.pone.0328625

**Published:** 2025-07-21

**Authors:** Quyuan Wang, Xinyu Ni, Yuping Tu, Ying Wang, Jiadi Liu

**Affiliations:** 1 Chongqing Key Laboratory of Intelligent Perception and BlockChain Technology, Chongqing Technology and Business University, Chongqing, China; 2 College of Computer and Information Science, College of Software, Southwest University, Chongqing, China; Beihang University, CHINA

## Abstract

The rapid development of edge computing and artificial intelligence has brought growing interest in collaborative training. While prior research has addressed technical aspects of resource allocation, less attention has been paid to the underlying economic mechanisms of resource trading. In this study, we examine how task publishers can effectively allocate budgets between computational and data resources during co-training. To address the uncertainty in data acquisition, we introduce Constant Proportion Portfolio Investment approach to assist in the construction of the payoff maximization problem with budget constraints. With the aid of economic tools, we design Swing Gradient Search Algorithm to obtain the optimal investment portfolio strategy, thereby addressing the coupling relationship between the quantities of resource acquisition. We also explore how market dynamics evolve in response to changes in supply and demand. To maintain dynamic market equilibrium, we develop two types of pricing algorithms, one based on stepped price adjustments for selected sellers, and another based on smoothed adjustments for all sellers. Simulation results demonstrate that the proposed strategies and algorithms offer acceptable performance in terms of algorithmic efficiency and strategic effectiveness, while also preserving fundamental economic principles and supporting stable market dynamics.

## Introduction

Edge Computing (EC) is becoming increasingly integrated with Artificial Intelligence (AI), and Federated Learning (FL) is a typical application paradigm that integrates AI tasks after splitting them up to be done collaboratively by edge users, with the expectation of reducing task cost and improving task performance [1–5]. Most current work focuses on how tasks are assigned, how they are aggregated, and the security of the processing.

This work focuses more on the economic ecosystem of collaborative training. For the edge users who are capable of handling the relevant tasks, they are actually contributing their surplus computational, storage, and data resources. One key issue that is often overlooked is the motivation of edge users to contribute resources, often referred to as the incentive problem. We analyze the motivations of task publishers and edge users from an economic behavioral perspective, where edge users profit from selling excess resources, and publishers of co-training tasks ensure task completion and their task performance by purchasing computational and data resources. However, the task publisher’s budget is often limited, so it is important to explore how the task publisher makes budget allocation, i.e., to explore how much money the task publisher spends on purchasing resources and what resources the task publisher chooses to purchases.

In terms of the co-training task, adequate data resources and computing resources are the prerequisite for completing a task. Therefore, in this paper, we focus on the problem of investing in a combination of these two resources. In traditional computing tasks, if the relevant resources are purchased the benefits are gained, but due to the specificity of the AI training task, the purchase of data resources does not necessarily result in a positive improvement in task performance, so we define data resources as risky resources, in contrast to computation resources, which are stable in terms of their improvement in task performance, and are therefore defined as risk-free resources. In summary, we further focus on how to allocate budgets when resources have risk attributes. We introduce the Constant Proportion Portfolio Investment (CPPI) theory to characterize the investment relationship between the two resources. In short, from a mathematical perspective, under the CPPI investment model, the investment amount in risky resources lies between a multiple of the return from risk-free resources and the remaining budget.

In economic behavior, we also need to pay attention to changes in the demand for resources in order to maintain market equilibrium, which refers to a dynamic stable state of demand and supply. One of the notable tools that can regulate market demand is the pricing mechanism, which can be used to raise the price of a resource when it is in short supply, both to protect the seller’s profits and to maintain market stability, and vice versa. We focus on two types of price adjustment mechanisms, one for stepped pricing adjustments for some sellers, and another for dynamically changing pricing adjustments for all sellers. In this paper, we first discuss the budget allocation strategy in the case of price stabilization, and then discuss how prices can be varied, which as a whole constitutes a set of dynamic equilibrium resource trading strategies.

Overall, in a collaborative training market for task publishers with the goal of maximizing their revenue, careful consideration needs to be given to how the task publisher allocates his budget on risk-free computational resources and risky data resources under a limited budget constraint. At the same time, reasonable resource price setting can also effectively ensure the revenue of resource providers and incentivize more edge users to participate in co-training tasks. To summarize, our contributions are summarized as follows.

- We construct a generalized transaction model of a collaborative training market where task publishers have limited budgets and resource providers sell computational and data resources. In particular, we utilize the Constant Proportion Portfolio Investment theory in microeconomics to portray the investment relationship between risky and risk-free resources. We carve out the payoff maximization problem for task publishers and decompose the problem into two parts.
- In the case of price stabilization, we consider how the budget is allocated between the two resources, and we use economic tools and theoretical calculations to drastically reduce the algorithmic search space. Since investments in risky and risk-free resources are coupled, we design a Swing Gradient Search Algorithm to solve optimization problems with coupled variables to obtain the optimal budget allocation strategy.
- To accurately reflect economic behavior in markets, we utilize microeconomic theory to reveal the dynamic coupling between demand fluctuations and resource price. Specifically, we propose three pricing algorithms: the Scale-based Pricing Algorithm (SPA), the Price Drop-based Pricing Algorithm (PDPA), and the Two-factor Adaptive Pricing Algorithm (TAPA). The first algorithm adjusts prices based on cluster size, while the latter two adjust prices in response to demand fluctuations, aiming to achieve market equilibrium.
- We have conducted a number of simulation experiments for the proposed algorithm and model, mainly comparing the performance of the algorithm in terms of algorithm efficiency and strategy effectiveness. In terms of algorithm efficiency, our budget allocation algorithm has a significant advantage over existing work due to the drastically reduced search space, and our price regulation algorithm has a simple structure with low time complexity. In terms of strategy effectiveness, our proposed algorithms all ensure the maximization of payoffs, while verifying the universal economic laws of the co-training market.

The rest of the paper is organized as follows. Section ‘Related Work’ reviews related work. In section ‘System Model’, we portray the various mathematical models used in this paper. Section ‘Budget Allocation Strategy’ focuses on the discussion of budget allocation algorithms, while section ‘Price Regulation Strategy’ analyzes price adjustment strategies. Relevant experimental results are shown in section ‘Numerical Simulation’.

## Related work

Our work was first inspired by the problem of resource allocation in edge computing and federal learning [6–9]. The authors propose a resource allocation algorithm to determine the bandwidth allocation, transmission power, and CPU frequency to minimize a weighted combination of total energy consumption, completion time and model accuracy in federated learning [10]. And a DRL-based resource allocation algorithm is proposed for Blockchain-Based Federated Learning to make the trade-off between the energy consumption and the convergence rate of the federated learning model by Zhang *et al*. [11]. Hou *et al*. utilize resource allocation to minimize the error bound with time and energy consumption budgets in federated learning [12]. Salh *et al*. focus on energy-efficient bandwidth allocation, CPU frequency, optimization transmission power, and the desired level of learning accuracy to minimize the energy consumption and satisfy the time requirement [13]. These works give us ideas in terms of research tools and methods, but they lack some economic perspective on the interactive behavior of resource transactions.

When it comes to resource trading behavior, we focus more on incentive design, budget allocation and pricing algorithms in edge computing and federal learning [14, 15, 17–23]. Li *et al*. investigate pricing mechanisms in federal learning, focusing on auction scenarios with one data seller and some data-limited entities as buyers. They verified the truthfulness, social welfare, and data sellers’ profit of the proposed mechanism [21]. Sun *et al*. introduce the TiFLCS-MAR Pricing Framework to maximize profitability for both clients and servers by leveraging Nash equilibrium principles for federated learning in data markets [22]. In the work, the Reputation-aware Opportunistic Budget Optimization approach for Auction-based Federated Learning are proposed to make the trade-off between saving costs and achieving target model performance [24]. And Liu *et al*. proposed a multitask data collection with a limited budget (MDCB) problem in edge computing and design the global maximum data first search algorithm and task sequence-based genetic algorithm to solve the problem [25].

This work incorporates volatility-based pricing strategies, which are inspired by the following studies. While volatility-oriented regulation has been explored in finance and supply chain, this study places greater emphasis on pricing mechanisms that respond to demand fluctuations [26–29]. Chen *et al*. proposed a dynamic pricing model considering the fluctuation in the day-ahead market [26]. Wang *et al*. optimized coordinated pricing strategies for trading and shared energy storage leasing in [27]. Inspired by these studies, the proposed pricing mechanism focuses on the interaction between demand fluctuations and price. And some studies have focused on the issue of price collusion in multi-agent collaboration, although beyond the scope of this paper, which provides valuable direction for future research [30–32].

On the basis of the above work, we focus on the impact of resource risk on profit maximization and discuss how to develop portfolio strategies when resources are risky, while focusing on the role of price adjustments in regulating the dynamic equilibrium of markets.

## System model

In this section, we introduce our basic models, including a trading model, a utility model, and a cost model. Then based on the above models the task publisher’s payoff is portrayed and the optimization problem is proposed.

### Trading model

**Fig 1:**
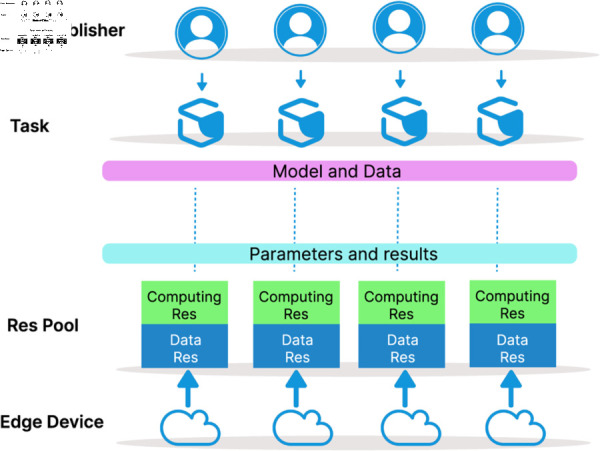
Illustration of collaborative training market player composition.

We consider three main roles in the proposed collaborative training market: task publishers, the resource pool, and edge devices. The task publisher purchases the required computing and data resources based on task attributes and requirements, and provides a base model and data for collaborative training. Edge devices offer the corresponding resources and return the final model parameters and training results. The overall system architecture is illustrated in Fig 1.

In a collaborative training market inspired by crowdsourcing, we assumed that the task publisher enhance the training performance by purchasing appropriate resources from edge devices. Thus, in general, within the market we consider, the task publisher is the only buyer, and edge devices are the sellers are sellers with appropriate resources. In this paper, the task publisher is denoted by $𝒬=\{Q_{1},Q_{2},...,Q_{J}\}$ , where *J* is the number of task publishers. Similarly we denote the set of edge devices by $ℰ=\{E_{1},E_{2},...,E_{I}\}$, where *I* is the number of available edge devices.

In the collaborative training market, as exemplified by the federated learning task, the performance of the co-training is closely related to the computation capacity and data of the edge devices. Therefore, in this paper, we focus on the trading of these two resources. First, intuitively, the edge device *i* provides computation resources to the task publisher *j* so that the task can be computed properly, and since the investment of computation capacity is proportional to the training performance (in general, the more computation resources invested, the better the training performance), the computation resources are also known as the risk-free resources. $N_{i}^{c}$ represents the number of computation resources available to *i*, and $p_{i}^{c}$ means the corresponding price of unit computation resources. We assume that each share of computing resources corresponds to the same amount of computation capacity *f* , and the upper bound of computation capacity that the edge device *i* can provide is $fN_{i}^{c}$.

In addition to this, the sample data used for training is also critical to the training performance, which we refer to as data resources in this paper. The edge device *i* itself has a certain number of training samples that belong to him, and we assume that the task publisher *j* already pays for these default data resources when he purchases the computation resources. To improve training performance, the task publisher can also purchase data resources from other edge devices and transmit them to the device performing the computation. We denote the price of data resources as *p*_*d*_ and the amount of data purchased by *j* is $n_{j}^{d}$. It is worth noting that larger amounts of data tend to lead to better training results, but this is not always the case, e.g., large amounts of redundant data do not effectively improve training performance, thus, the data resources are also known as the risk resources.

For each task publisher *j*, we define his maximum budget as *b*_*j*_, which is the maximum amount that the task publisher *j* is willing to invest in this task. The task publisher can allocate their budgets based on expected utility and their own type of risk tolerance.

### Utility model

For the task publisher of collaborative training network, the utility comes from two main sources, the time-saving gains from distributing the task, and the gain in training performance from the fresh data provided by the edge intelligent agents, which we refer to as the data gain.

We assume that both the computation time and transmission time are related to the amount of data being computed (or transmitted). Similar to related works [34, 44, 45], in order to highlight the contributions of this paper at the strategic level, we simplify the computation and communication processes. Specifically, the time-savings gains refers to the economic benefit of the time saved by the execution of the task publisher when purchasing computational and data resources from the edge device, and the gains $u_{j}^{t}$ are expressed as follows:

$$u_{j}^{t}=(T_{j}-\frac{o_{j}+n_{j}^{d}*d}{n_{j}^{c}f}-\frac{o_{j}+n_{j}^{d}*d}{r_{net}})\gamma_{t},$$ (1)

where $n_{j}^{c}=\sum_{i}n_{j,i\;}^{c}$, $n_{j}^{d}=\sum_{i}n_{j,i\;}^{d}$, and $n_{j,i\;}^{c}$ portrays the number of computing resources purchased by task publisher *j* from edge device *i*. The $\gamma_{t}$ is the economic benefits per unit of time, *T*_*j*_ denotes the maximum execution time that task publisher *j* can tolerate. *o*_*j*_ represents the amount of original data owned by the task publisher, while $n_{j}^{d}$ represents the amount of additional data purchased by the task publisher, and *d* is the amount of data contained per unit of data resource. *r*_*net*_ denotes the resource transmission speed within the network.

According to the relevant literature [33, 34], data-level gains are mainly closely related to the amount of data used by edge devices for training. Generally speaking, the data gain increases as the amount of data increases, but the marginal increase decreases with redundant data, etc. We utilize the following form to portray the data gain:

$$u_{j}^{d}=d*n_{j}^{d}(1-\alpha_{i}e^{-\theta_{i}})\gamma_{d},$$ (2)

where $\gamma_{d}$ is the economic benefits per unit of data contribution. $\alpha_{i}(>0)$ and $\theta_{i}(>0)$ are constant coefficients. $\alpha_{i}$ is called the data fluctuation factor that characterizes the negative impact on the training effectiveness of edge device *i* as the amount of data increases, and the larger $\alpha_{i}$ is the lower the data contribution is. $\theta_{i}$ is called the data increment factor, which characterizes the positive impact of edge device *i* as the amount of data increases.

### Cost model

From a microeconomics perspective, task publishers spend their budgets to purchase two resources that are essentially investment behaviors. In this paper, computational resources are a mandatory requirement for collaborative training, while data resources are optional. Furthermore, computational resources are relatively low-risk, low-return resources. Compared to data resources, the impact of computational resources on training outcomes is predictable. On the other hand, data resources can yield unexpected surprises or deficiencies due to variations in data quality. The option we offer to task publishers is to purchase additional training data (some free training data is provided by edge devices). However, due to the privacy nature of the data, it is impossible to accurately assess the quality of the data, making data resources relatively high-risk, high-return. The cost of purchasing computation and data resources are as follows:

$$c_{j}^{c}=\sum_{i}n_{j,i\;}^{c}p_{i}^{c}$$ (3)

$$c_{j}^{d}=\sum_{i}n_{j,i\;}^{d}p_{i}^{d}$$ (4)

The weighting of the task publisher’s portfolio towards the two resources will deviate from the original investment strategy objectives over time and under resource price changes, with the possibility of excessive portfolio risk. Through re-balancing, the portfolio will return to its target allocation to match the investor’s initial investment objectives and risk profile. Therefore, we introduce the Constant Proportion Portfolio Investment (CPPI) to assist in portraying the financial investment made by task publishers in purchasing these two resources [35].

There are several key metrics in the CPPI strategy, *Cushion* represents the difference between the current value brought by the portfolio and the minimum amount of protection (*Floor*), and *Multiplier* represents the amount of risk that the investor is willing to carry. To adapt in the collaborative training market, we set the key parameters as follows:

$$Cushion=u_{j,i\;}^{t}-c_{j,i\;}^{c}=u_{j,i\;}^{t}-n_{j,i\;}^{c}\times p_{i}^{c},$$ (5)

where the cushion specifically represents the payoff generated from the purchase of computational resources, and the floor is the amount of money spent on purchasing computational resources.

$$c_{j,i\;}^{d}=min\{\eta_{j}Cushion,b_{j}-c_{j,i\;}^{c}\},$$ (6)

where $\eta_{j}$ is the multiplier to highlight the level of risk the task publisher is willing to take.

From the mathematical expression, the total investment of the task publisher in purchasing a high-risk resources such as the data resources is the minimum of the multiplier multiplied by the cushion (actual payoff on the low-risk resource) and the budget remaining after purchasing the low-risk resource, which also guarantees that the total budget cannot be overspent.

### Problem formulation

The perspective of this paper is to safeguard the revenue of edge devices in the collaborative training market while maximizing the payoffs for task publishers to make it worth their while. On the one hand, the task publisher benefits from the utility gains obtained by purchasing the relevant resources, and on the other hand, he benefits from the budget surplus. We characterize the task publisher’s payoff as a weighted sum form of utility gain and residual budget as follows:

$$P_{j}=u_{j}^{t}+u_{j}^{d}-c_{j}^{c}-c_{j}^{d}+\beta_{j}(b_{j}-c_{j}^{c}-c_{j}^{d}),$$ (7)

where $\beta_{j}$ is the equilibrium factor between utility gain and budget surplus for task publisher *j*, reflecting the importance of direct payoffs on *j*. Then the the payoff maximization problem can be formulated as:

$$OPT-1:\;\max_{n,p}P$$ (8)

subject to $\forall T_{j}\in 𝒯,\forall E_{i}\in ℰ.$


$$\text{C1:}\;\sum_{T_{j}\in T}n_{j,i\;}^{c}\leq N_{i}^{c},\forall E_{i}\in ℰ,$$

$$\text{C2:}\;p_{i}^{c}\geq 0,p_{i}^{d}\geq 0,\forall E_{i}\in ℰ$$

$$\text{C3:}\;u_{j,i\;}^{t}\geq 0,u_{j,i\;}^{d}\geq 0,\forall T_{j}\in 𝒯,\forall E_{i}\in ℰ.$$

## Budget allocation strategy

We focus in this section on how task publishers should allocate their budgets when market prices are stable.

### Boundaries of the strategy

As introduced earlier, there are two commodities in the collaborative training market: risk-free resources (computation capacity) and risky resources (data). Correspondingly, there are also two types of task publishers (resource buyers), the conservative ones who are completely unable to take risks and the aggressive ones who prefer high risks.

First, whatever budget allocation strategy the task publisher has, it should satisfy constraint C3 in **OPT-1**, that is the gain from purchasing the resource should be greater than 0. For the conservatives, their willingness to take risk is 0, i.e. $\eta_{j}=0$. They will only buy the computation capacity resources, therefore, we make Eq 1 equal to 0, and we can derive the minimum amount of computation capacity to be purchased in the case of satisfying constraint C3, which is a **lower bound** for the budget allocation strategy:

$$T_{j}-\frac{o_{j}}{n_{j,i\;}^{c}f}-\frac{o_{j}}{r_{j,i\;}}=0\;\Rightarrow n_{j,min}^{f}=\frac{o_{j}}{(T_{j}-\frac{o_{j}}{r_{max}})f}.$$ (9)

This lower bound ensures that co-training tasks are completed within the time boundaries tolerated by the task publisher. In addition, since no data resources will be purchased, the total payoff to the conservative can be rewritten as:

$$P_{j}^{con}=u_{j}^{t}-c_{j}^{c}+\beta_{j}(b_{j}-c_{j}^{c}).$$ (10)

Accordingly, the objective function of payoff maximization problem **OPT-1** is reconstructed as:

$$OPT-2:\;\max_{n_{j}^{f}}P_{j}^{con}=\max_{n_{j}^{f}}u_{j}^{t}-c_{j}^{c}+\beta_{j}(b_{j}-c_{j}^{c})$$ (11)

By substituting the relevant formulas and parameters, we can find the optimal value of computation capacity resource purchase for the conservative strategy:

$$n_{j}^{f*}=\sqrt{\frac{o_{j}\gamma_{t}}{(1+\beta_{j}){\hat{p}}_{f}f}},$$ (12)

which is the **computation capacity upper bound** for the budget allocation strategy.

Secondly, we consider another special case, for aggressive ones, according to the Eq 6, when $\eta_{j}=\infty$, they are willing to invest all of the budget left over from the purchase of computational resources in data resources. At this point, the task publisher spends all of his budget on purchasing these two resources, and in economic terms, such a curve is called the iso-cost line, which describes the quantitative relationship between the purchase of the two resources under a limited budget. All eligible purchase portfolios should be in the area below this line.

**Fig 2:**
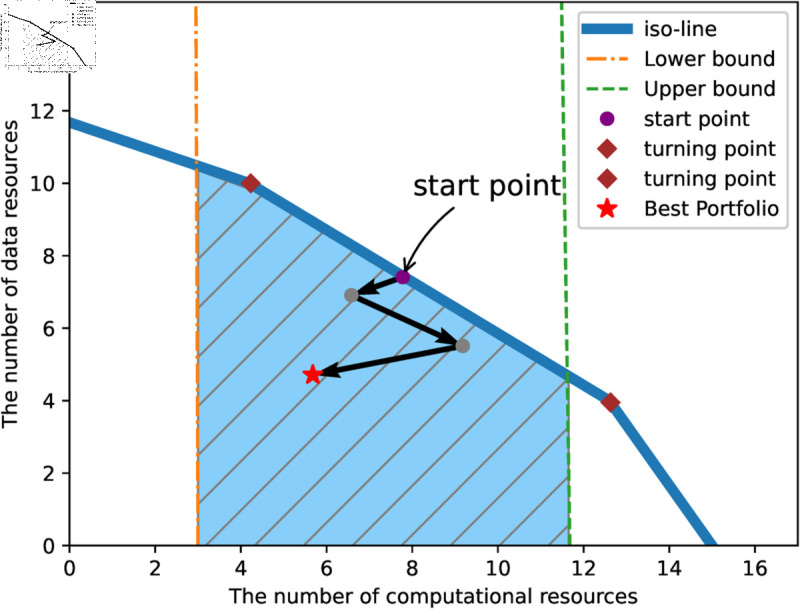
The illustration of three boundaries.

Therefore, we utilize the lower bound, the computation capacity upper bound, and the iso-cost line to achieve the purpose of narrowing the policy search area, and Fig 2 visualizes the role of the three boundaries.

Fig 2 represents an illustrative case only, where the shaded area is the actual search area of the budget allocation strategy, and the feasible portfolio strategy is located within the area bounded by the upper and lower boundaries and the iso-cost line. It is worth noting that the iso-cost line is not a line with a fixed slope, but rather a patchwork of multiple slopes whose slopes are the ratio of the prices of the two resources. The intersection of the iso-cost line with the horizontal and vertical axes represents the amount of the resource that can be purchased with the entire budget, and the inflection point means that the current seller has sold out of the resource.

### Swing gradient search algorithm for portfolio strategy

After narrowing the search area, the corresponding budget allocation algorithm can be designed to find the optimal strategy. However, there are still two issues that need to be addressed in this specific scenario.

Firstly, by observing Eq 12 and Eq 6, we can find that there is an optimal numerical solution for the quantity of computation resources when we are given the quantity of data resources. However, since data resources are a risky investment, the purchase volume also depends on the amount of computational resources and the expected returns. Therefore, we consider that there is a coupling relationship between the two.

Therefore, we adopt a nested double-iteration approach for decoupling. **In the inner loop**, we set a data resource purchase quantity $n^{d_{1}}$, we assume that the average price is the average price of all the resources sold, and accordingly calculate the computation resource purchase quantity $n^{c_{1}}$ in the current state, and then update the average price, and then accordingly calculate the computation resource purchase quantity $n^{c_{2}}$, until the average price (or the number of computation resources) is no longer changing or the range of change is sufficiently small, and at this point the computation resource purchase quantity is $n^{c_{1}^{*}}$, and $\left\{n^{d_{1}},n^{f_{c}^{*}}\right\}$ is the portfolio investment strategy in the current state. This process, which we call **swing**, corresponds to lines 12-18 in Algorithm 1.

**In the outer loop**, we follow the gradient descent method to obtain the next data resource quantity $n^{d_{2}}$, for each data resource purchase, we obtain the computation resource quantity in the current situation by the above method until the gradient descent of the outer layer converges, and the final optimal portfolio investment strategy is $\left\{n^{d*},n^{c*}\right\}$.

The second issue we need to focus on is the algorithm’s initial point. In finding the initial point we follow two rules. **Rule 1**: The starting point is set on the iso-cost line within the feasible region. Regardless of the type of task publisher, rational people will tend to buy as many relevant resources as possible to improve performance, and the strategies on the iso-cost line are all budget exhaustion cases, which are theoretically closer to the optimal strategy. **Rule 2**: We choose the midpoint of the feasible region as the starting point. Since the algorithm has a left-right swing solution process, in order to avoid ending the optimization process by touching the boundary by mistake. In summary, we choose the midpoint of the iso-cost line in the feasible region as the initial point of the algorithm, and the specific algorithm process is shown in Algorithm 1. In the worst case, the complexity of Algorithm SGSA is $Iter_{max}*NF$, where *NF* is the number of sellers of computation resources.

**Algorithm 1 Swing Gradient Search Algorithm (SGSA).**

**Input:** iso-cost line *L*_*j*_, lower bound $n_{j,min}^{c}$, upper bound $n_{j}^{c*}$,

resource price and quantity, risk coefficient $\eta_{j}$.

**Output:** Optimal portfolio strategy [${\text{n}}^{\text{d}*},{\text{n}}^{\text{c}*}$].

1: Construct the search area based on three boundaries.

2: **if** No feasible search area **then**

3:   **return** No optimal portfolio.

4: **end if**

5: Find a portfolio strategy on ${\text{n}}^{\text{d}},{\text{n}}^{\text{c}}$ the iso-cost line *L*_*j*_

6: Calculate the average price $\hat{p_{f}}$ of computing resource.

7: Calculate $\hat{n^{c}}$ based on ${\text{n}}^{\text{d}}$ and $\hat{p_{f}}$.

8: **while** $m_{g}\leq Iter_{max}$ and loc = True **do**

9:   Find the ${\text{n}}^{\text{d}*}$ using Gradient Method.

10:   *m* = *m* + 1

11:   **if** location of the portfolio is in the feasible search

area **then**

12:    **while** $n^{c}\neq \hat{n^{c}}$ and $m_{l}\leq NF$ **do**

13:     Calculate the next price ${\hat{p_{f}}}^{\prime }$ based on $\hat{n^{c}}$.

14:     Set $\hat{p_{f}}={\hat{p_{f}}}^{\prime }$.

15:     Calculate the next computing resource $n^{c^{\prime }}$ based on

price $\hat{p_{f}}$.

16:     Set $n^{c}=\hat{n^{c}}$

17:     Set $\hat{n^{c}}=n^{c^{\prime }}$

18:    **end while**

19:    loc = False

20:   **end if**

21: **end while**

22: **return** Optimal portfolio strategy.

In this section we have addressed the task publisher’s budget allocation problem, i.e., the problem of how to maximize the benefits of purchasing relevant resources with a limited budget, but this problem presupposes that the market is in a steady state, specifically, that the price of the resource is stable. Therefore, in the next section we will focus on how the price move to drive the market to equilibrium.

## Price regulation strategy

In this section, we focus on how resource providers adjust their resource prices based on current market conditions. Microeconomics suggests that the price of a commodity is closely related to the supply and demand in a market, thus we first define what supply and demand are in the co-training market, respectively.

In the co-training market, the resource provider is the edge device that provides data or computing capacity, and the buyer is the task publisher. We consider a task publisher to be a potential buyer only if his disposable budget is greater than 0. Therefore, we need to further define potential buyers as follows:

**Definition 1** (Potential Buyer). *The task publisher Q_j_ is denoted as a potential buyer if and only if his disposable budget b_j_>0. A set of all potential buyer is ${𝒬}_{p}$.*

In microeconomics, supply and demand are generally intuitively reflected in the quantities of goods supplied and purchased. In this paper, we multiply the quantities by their prices. Therefore, supply is represented as the sum of the number of resources offered in the system multiplied by their price, and demand is represented as the amount spent by the task publisher. We have the following definitions:

**Definition 2** (Supply). *The sum of quantity of resources multiplied by price in the market.*

$$ℛ=\sum_{{ℰ}_{i}}(N_{i}^{c}p_{i}^{c})+\sum_{{ℰ}_{k}}(N_{k}^{d}p_{k}^{d}),$$ (13)

**Definition 3** (Demand). *The amount that task publishers are willing to spend based on the budget allocation strategy.*

$$𝒟=\sum_{Q_{j}\in {𝒬}_{p}}(c_{j}^{d}+c_{j}^{c}).$$ (14)

Typically, economists take the state of market clearing to judge whether the current market has reached equilibrium, and in this paper we consider the market to be in equilibrium when sellers have sold out of resources while buyers have spent what they wanted to spend, and we have the following definition:

**Definition 4** (Market Equilibrium). *When ℛ=𝒟, we assume that the co-training market reaches equilibrium.*

We can determine the current market state by the difference between supply and demand, and this market state generally reflects the reasonableness of the resource price by the resource provider. For example, when supply exceeds demand and task publishers are unwilling to buy the resource concerned, it might be that the resource is overpriced, and thus the resource provider will reduce the price appropriately, and vice versa. We then design the price regulation strategies based on this overall idea to bring the co-training market to equilibrium.

### Object-oriented price regulation strategy

Based on market observation and life experience, consumers mostly choose head brands when purchasing goods. By head, we mean the most popular suppliers. Therefore, we design the first price adjustment algorithm, Scale-based pricing algorithm (SPA), to scale for these most popular suppliers.

Algorithm 2 specifically shows the process flow of SPA. As mentioned earlier, we use the demand-supply difference to portray the popularity of resource providers based on the microeconomics theory [38–40]. We rank the suppliers using the calculated demand-supply gap and build the set of potential suppliers based on the scale coefficient, and subsequently adjust the resource prices for the suppliers in the set, and when the market is in equilibrium, we obtain the optimal resource prices.

**Algorithm 2 Scale-based pricing algorithm (SPA).**

**Input:** Market supply ℛ, market demand 𝒟, resource price, scale

coefficient ε, price adjustment step $\Delta_{p}$

**Output:** Resource prices when market equilibrium ${\text{p}}^{\text{c}*}$, ${\text{p}}^{\text{d}*}$

1: Calculate the demand-supply differences for each supplier

𝒟−ℛ

2: **if** 𝒟−ℛ>0 **then**

3:   Ranking the resource suppliers in descending order of the

difference

4:   List of top ε suppliers of potential suppliers

5: **end if**

6: **while** Market equilibrium not reached **and** supplier in

potential suppliers **do**

7:   Update resources price $p_{new}=p_{old}+\Delta_{p}$

8: **end while**

9: **return** Resource prices when market equilibrium ${\text{p}}^{\text{c}*}$, ${\text{p}}^{\text{d}*}$

Theoretically, the setting of the scale coefficient is beneficial for fine-tuning the market, and price adjustments for key suppliers are beneficial for market efficiency. However, in terms of algorithmic efficiency, the larger the scale coefficient, the lower the complexity of the algorithm, and the faster the algorithm converges. In the worst case, the time complexity is $O(𝒟/(\varepsilon *\Delta_{p}))$.

In Algorithm 2, we focus on the object of price regulation, we simply specify the update step of the price through the parameter setting, and then we are going to concentrate on how to set the strategy of price update.

### Volatility-oriented price regulation strategy

In real life we can indeed determine whether to increase or decrease prices based on the state of market demand, however, if all sellers increase prices by a large amount (a secret alliance to maximize profits), consumer willingness to consume will be drastically reduced, and the new market equilibrium will be that there is no buying or selling, which is something we don’t expect to happen, thus we have to control the strategy of price changes based on the state of the market.

In this case, we propose a price regulation algorithm based on the drop, the mentioned drop refers to the difference between the highest selling price and the lowest selling price of a certain resource in the current market state, and the price control step of each eligible supplier is related to this drop, so as to ensure that the price amplitude is controllable. Algorithm 3 demonstrates this idea. In short, unlike the previous algorithm, each supplier that meets the requirements should have its price adjusted by a margin that is tightly correlated to the price drop in the current state.

**Algorithm 3 Price drop-based pricing algorithm (PDPA).**

**Input:** Market supply ℛ, market demand 𝒟, resource price,

highest price *p*_*max*_, lowest price*p*_*min*_, drop coefficient ξ

**Output:** Resource prices when market equilibrium ${\text{p}}^{\text{c}*}$, ${\text{p}}^{\text{d}*}$

1: **while** Market equilibrium not reached **do**

2:   Calculate the demand-supply differences for each supplier

𝒟−ℛ

3:   **if** 𝒟−ℛ>0 **then**

4:    Update resources price $p_{new}=p_{old}+\xi (p_{max}-p_{min})$

5:   **else**

6:    Update resources price $p_{new}=p_{old}-\xi (p_{max}-p_{min})$

7:   **end if**

8: **end while**

9: **return** Resource prices when market equilibrium ${\text{p}}^{\text{c}*}$, ${\text{p}}^{\text{d}*}$

However, while the above algorithm specifies the magnitude of the price change, it does not specify the range of prices, and it is still possible for the adjusted price to be too high or too low, and this is also closely related to the number of iterations.

In principal, there are multiple interventions in a well-developed price regulation strategy. For example, on the one hand, when the gap between demand and supply is too large, the magnitude of price regulation should be larger. On the other hand, if market equilibrium has not been reached after many attempts at regulation, the magnitude of price regulation should be moderately increased. Thus, mathematically, we define two-factor adaptive price volatility as follows:

$$\Delta_{p}=\frac{\upsilon^{2}(𝒟-ℛ)+\tau^{2}\rho }{\upsilon \tau },$$ (15)

where υ and τ are two-factor adaptive coefficients, and ρ is the current iteration number. Based on this price variation strategy, we propose the two-factor adaptive pricing algorithm (TAPA), the specific process is shown in Algorithm 4.

**Algorithm 4 Two-factor adaptive pricing algorithm (TAPA).**

**Input:** Market supply ℛ, market demand 𝒟, resource price,

maximum affordable price *p*_*max*_, maximum single adjustment

*p*_*s*,*max*_, minimum single adjustment *p*_*s*,*min*_ two-factor adaptive

coefficients υ and τ, maximum number of iterations $\rho_{max}$.

**Output:** Resource prices when market equilibrium ${\text{p}}^{\text{c}*}$, ${\text{p}}^{\text{d}*}$

1: **while** Market equilibrium not reached **and** $\rho <\rho_{max}$ **do**

2:   **for** All suppliers **do**

3:    Calculate the demand-supply differences for each

supplier 𝒟−ℛ

4:    **if** 𝒟−ℛ>0 **then**

5:     $\Delta_{p}=max\{p_{s,min},min\{p_{s,max},\Delta_{p}\}\}$

6:     $p_{new}=min\{p_{old}+\Delta_{p},p_{max}\}$

7:     Update resources price

8:    **end if**

9:   **end for**

10: **end while**

11: **return** Resource prices when market equilibrium ${\text{p}}^{\text{c}*}$, ${\text{p}}^{\text{d}*}$

In Algorithm 4, we mimic the stock market trading mechanism by setting a maximum and minimum value for each adjustment range, and limit the range of the final price to no more than the affordable range, while adjusting the magnitude of each price change according to Eq 15. Specifically, when price regulation first begins, the difference between demand and supply is large, so the demand gap dominates price regulation, and as the number of iterations increases, the influence of the number of iterations gradually increases. In worst case, the time complexity of Algorithm 4 is $O(𝒟/p_{s,min})$.

### Strategic interaction workflow among participants and mechanisms

**Fig 3:**
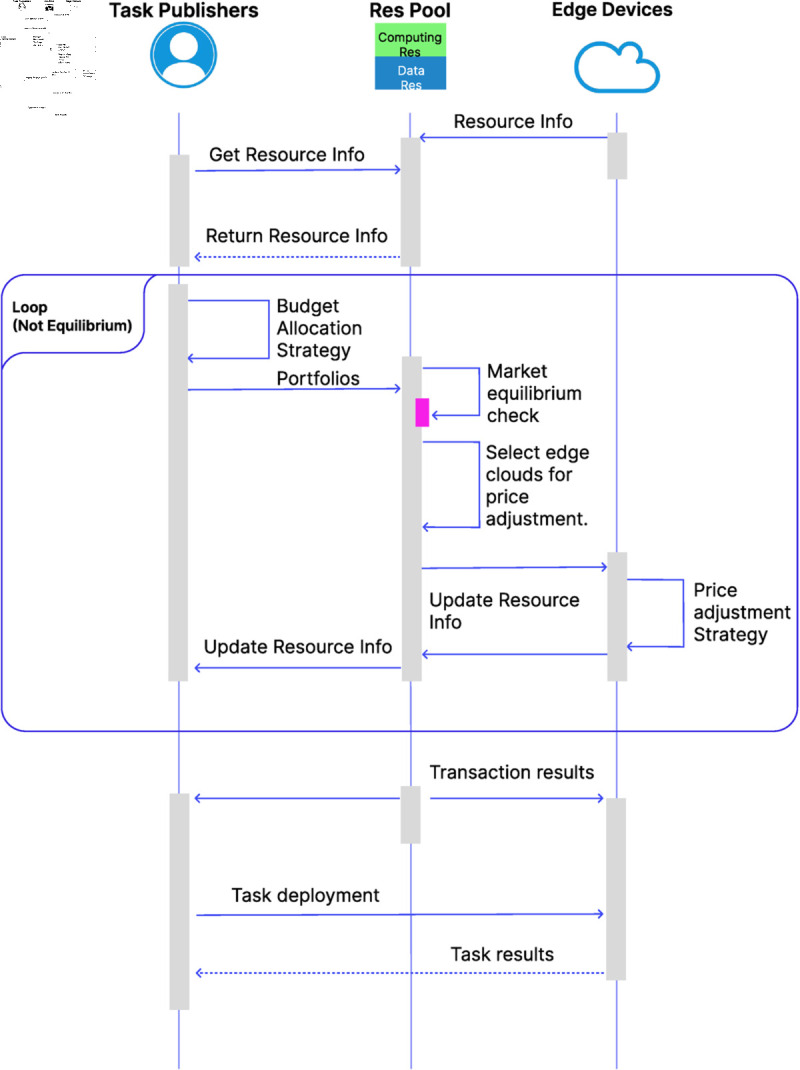
Strategic interaction workflow among participants and mechanisms.

After introducing the Budget Allocation Strategy and the Price Regulation Strategy, this subsection aims to illustrate how the two strategies interact during system operation, as well as how participants engage with one another within the system. A visual representation of the interaction process is provided in Fig 3 to support the explanation.

The interaction between edge devices and task publishers is based on the resource information maintained in the resource pool. Initially, edge devices submit their available computational and data resources to the resource pool. Task publishers then access this aggregated information to formulate their portfolio strategies. These strategies are submitted back to the resource pool for evaluation the current market state.

If the market is found to be out of equilibrium, it identifies a subset of edge devices whose resource prices require adjustment. These selected edge devices then update their prices according to the predefined price regulation strategy. The resource pool refreshes the resource information accordingly, and task publishers update their portfolio strategies based on the revised information.

This iterative process continues until the market reaches equilibrium. Once equilibrium is achieved, the resource pool finalizes the matching results and communicates the transaction outcomes to both edge devices and task publishers, enabling subsequent task deployment.

## Numerical simulation

In this section, we utilize numerical simulations to validate the effectiveness of the proposed strategy. The experiment involves four Dell R750XS servers deployed in different geographic locations. Each server features a 2U configuration with dual Intel Xeon Silver 4210 2.2GHz CPUs, 128GB RAM, a 1TB HDD, and a 2TB SSD. Using virtualization technology, we deploy two types of virtual edge devices on these servers: one with high-performance computing capacity and the other with high-quality data resources. To emulate a real-world scenario, we randomly generate edge devices and tasks based on specified parameters.

Through comparison with related work and preliminary experiments, the key parameter settings involved in the numerical simulation are as follows [41–43]. For task publishers, the payoff per unit time is set at 10, and the payoff per unit data at 50. Budgets range from a minimum of 20 to a maximum of 100, while execution times range from 5 to 30 units. For edge devices, computation capacities vary between 10 and 50, with computation resource prices ranging from 1 to 20. Data capacities range from 10 to 30, and data resource prices also span 5 to 20. Additional simulation parameters include a maximum iteration count of 200. Finally, we set the maximum iteration number as 200, the scale coefficient ε as 0.5, the drop coefficient ξ as 0.5, the two-factor adaptive coefficients υ and τ as 0.5, the price adjustment step $\Delta_{p}$ as 0.5. The main source code for our implementation is available at https://github.com/jiadiryu/CollabTrade-RiskAware.

### Algorithm efficiency

**Fig 4:**
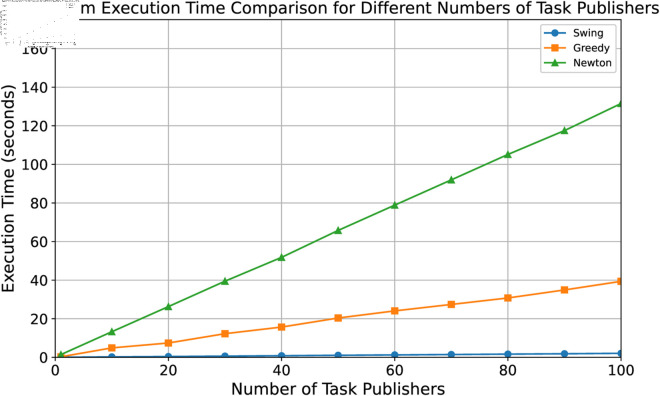
The execution time comparison.

To compare the time cost under different algorithms, we increase the number of task publishers from 10 to 100 to observe the time cost of the three algorithms. For each number setting of task publishers, we repeat the experiment 20 times and take the average value as the final result. In each time of experiment, we randomly generate task publishers according to our parameter settings, and keep the resource providers fixed. The task publishers need to run each algorithm to obtain the portfolio strategy, and we record the time cost of each algorithm. In the experiment, we mainly react to the algorithm’s efficiency by its execution time and the number of iterations when convergence is reached. Fig 4 shows the time required by the three algorithms to obtain the optimal portfolio strategy when prices are stable. Our proposed SGSA algorithm not only takes the shortest time, but also the execution time grows very smoothly and receives almost no effect from the number of task publishers. This is because we first constrain the search space when searching for an optimal strategy, and secondly our swing algorithm demonstrates an advantage in efficiency when fixing one side to find the optimal solution of the other.

**Fig 5:**
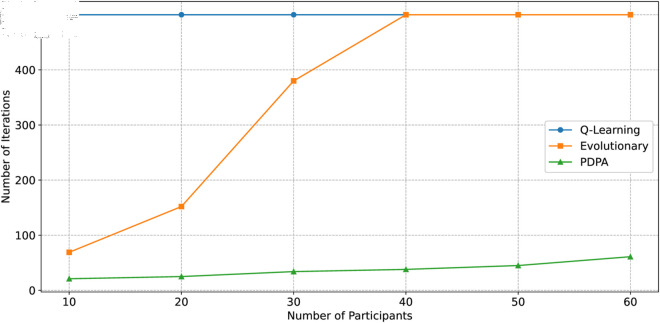
Comparison of convergence speed with other algorithms.

For the pricing algorithms, we compare it with Q-learning based algorithm [36] and evolutionary algorithm [37] in Fig 5. In this experiment, we set equal numbers of edge devices and task publishers as participants in the resource market, and we increase the number of participants from 10 to 60 to compare the performance approaches. We set the maximum iteration number as 500 for all approaches which as efficiency threshold of the market. For each number setting of participants, we randomly develop edge devices and task publishers and repeat 10 times to obtain the average results. Due to the existence of Q-table updates in Q-learning based algorithm, the number of iterations required to reach market equilibrium is high but relatively stable, and the convergence speed of evolution-based algorithms is significantly related to the number of participating individuals, with slower convergence speeds when the number of individuals is high. Our PDPA algorithm only needs to judge the seller’s supply and demand and then adjust the price, which has the lowest time complexity.

**Fig 6:**
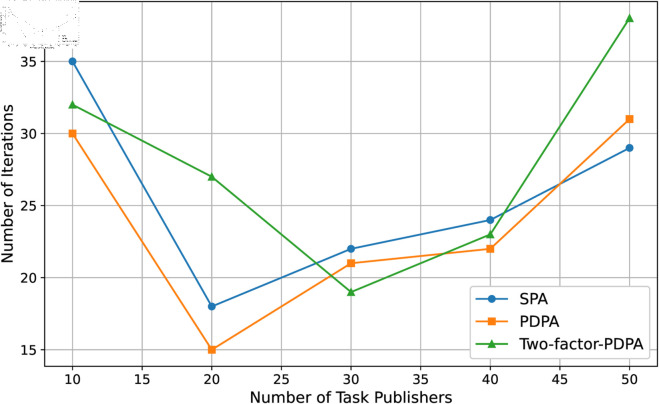
Demonstration of algorithm convergence.

**Fig 7:**
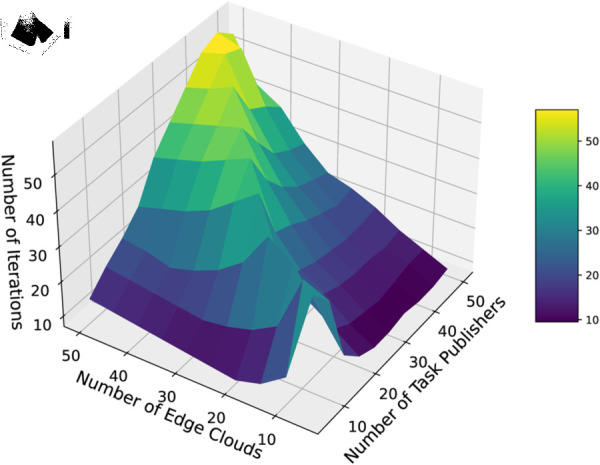
The algorithm convergence when the number of buyers and sellers varies.

In this experiment, we first set fixed numbers of edge devices and increase the number of task publisher to observe the iteration times of the algorithms to reach the equilibrium of the market. We repeat the experiment 10 times and take the average value as the final result which is shown in Fig 6. Then we set the number of task publisher and the number of edge devices from 10 to 60 to observe the convergence speed of the algorithms under different numbers of participants. The average results of the 10 times repeated experiment are shown in Fig 7. For each algorithm, there is a small fluctuation in its convergence, which is due to the fact that when the number of buyers and sellers is small, especially the number of sellers is small, there is a low supply of resources, and more iterations are needed to reach the market equilibrium. As the amount of resources increases, the convergence becomes faster, but if the number of individuals continues to increase, the competition becomes larger and the number of iterations required rises again.

### Strategy effectiveness

We compare the effectiveness of the proposed algorithm mainly in terms of the payoff and the price of the resources.

**Fig 8:**
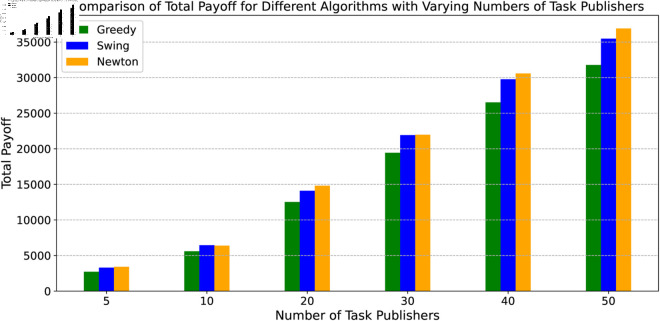
The comparison of payoff.

In this experiment, we develop 30 edge devices and the number of task publishers from 5 to 50 to observe the total return of each algorithm. In each process of experiment, we use the SGSA algorithm to obtain the optimal portfolio strategy for each task publisher, and then we use different pricing algorithms to calculate the total payoff of each task publisher. The average results of the 10 times repeated experiment are shown in Fig 8. In terms of total payoff, our proposed Swing algorithm lies between Newton method and the Greedy method, which is illustrated in Fig 7. This is because Newton method essentially guarantees the optimal solution and therefore brings the greatest payoff, however, combined with the speed of convergence, we have a large improvement in convergence speed and do not lose much at the payoff.

**Fig 9:**
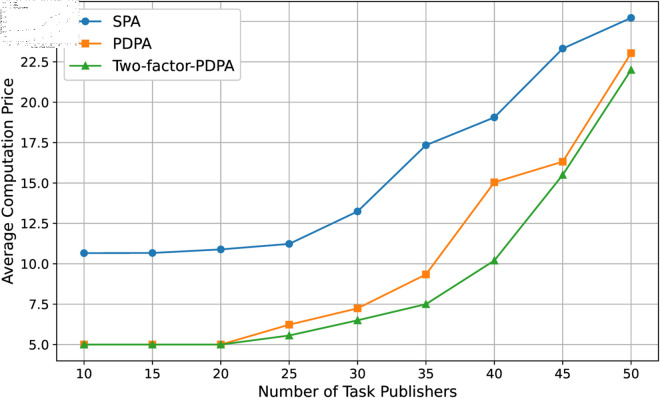
Computation resource price.

**Fig 10:**
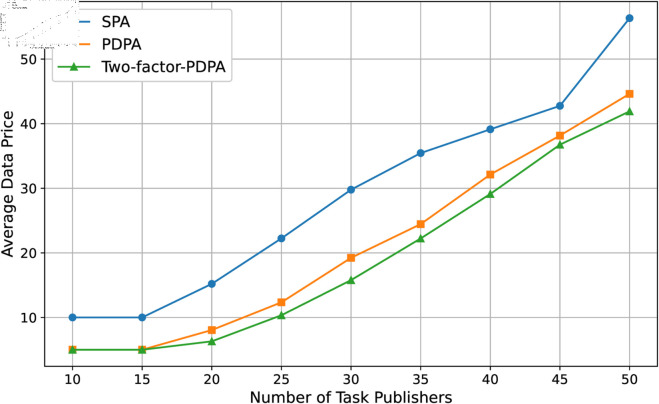
Data resource price.

We set the number of the task publisher from 10 to 50 to observe the change of average price of the two resources under different price regulation strategies. In each stage of experiment, we use the SGSA Algorithm for each task publisher to obtain the optimal portfolio strategy, and get the average price of the two resources through different pricing algorithms. The results are shown in Figs 9 and 10. Most notably, resource prices were relatively stable in the early stages when the number of resources was relatively saturated. However, as the number of tasks rises, the demand for both resources increases dramatically, and the average prices of the resources both rise accordingly, which reflects well the economic laws and fairness of the market.

Second, the relatively high average price of data resources implies that they are more important in our system setup, which of course depends on the parameter settings and is in line with what is currently common in the co-training market. Finally, as a result of a more rigorous price adjustment strategy, the Two-factor PDPA algorithm always gives the lowest price for the resource.

**Fig 11:**
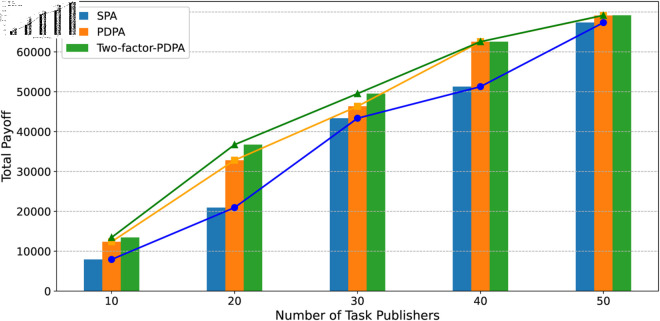
Changes in payoffs from pricing strategies.

In this experiment, we focus on the total payoff of each algorithm under different price regulation strategies. We use fixed edge devices and increase the number of task publishers from 10 to 50 to observe the change of total payoff of each algorithm. Each pricing algorithm is applied based on the same setting for portfolio strategy of each task publisher. The results are shown in Fig 11. Several pricing algorithms also bring significant payoff gains as the number of task publishers increases. It’s important to note that since the payoff is for the task publisher, the less money spent on purchasing resources, the higher the overall payoff, subject to completion of the task. In Fig 10, Two-factor PDPA delivers the highest total payoffs, which is due to a more precise price regulation strategy.

In summary, the algorithms we designed demonstrate promising performance in simulated scenarios of a certain scale, with both algorithmic efficiency (e.g., convergence behavior and runtime) and effectiveness (e.g., price stability and payoffs) falling within acceptable ranges. However, the proposed mechanism still faces challenges related to algorithmic complexity when applied to ultra-large-scale cluster environments.

## Conclusion

In this paper, we focus our discussion and research on resource trading behavior in collaborative training. When the price is relatively stable, we focus on how the limited budget can be allocated between two types of resources (risky and risk-less), and we draw on the tools of economics to narrow down the search space of the algorithm, while designing a Swing Gradient Search Algorithm to solve the coupling relationship between the purchases of the two resources and to find the optimal portfolio strategy. When trading behavior occurs in the market, supply and demand will change, and we hope to use the pricing mechanism to maintain the dynamic equilibrium, while ensuring that the market is reasonable and fair. Thus, we design two types of pricing algorithms, one implementing stepped price changes for all sellers and the other implementing smoothed price changes for some sellers. We verified the efficiency of our algorithms and the effectiveness of our strategies through simulation experiments, both of which showed advantages.

However, in this work, in order to design a lightweight and as general as possible resource trading model, we had to make certain assumptions, including assumptions in the modeling process and trade-offs in algorithm design. Therefore, to address the limitations identified in this work, we hope to make the following improvements: we aim to leverage machine learning, reinforcement learning, and other techniques to automatically select the initial point for the SGSA as well as to optimize the internal parameters of the pricing algorithms.

Regarding future work, we hope to further explore the issues of budget allocation and re-utilization in human-machine collaboration environments, which represent a new cooperative architecture receiving significant attention in the era of artificial intelligence. We also aim to explore the dynamic collaborative processes of information transmission, trust evolution, and strategy feedback among intelligent devices of different levels during long-term interactions.

## References

[1] Wang W, Kumar N, Chen J, Gong Z, Kong X, Wei W, et al. Realizing the potential of the Internet of Things for smart tourism with 5G and AI. IEEE Network. 2020;34(6):295–301. doi: 10.1109/mnet.011.2000250

[2] Abbas N, Zhang Y, Taherkordi A, Skeie T. Mobile edge computing: a survey. IEEE Internet Things J. 2018;5(1):450–65. doi: 10.1109/jiot.2017.2750180

[3] Li T, Sahu AK, Talwalkar A, Smith V. Federated learning: challenges, methods, and future directions. IEEE Signal Process Mag. 2020;37(3):50–60. doi: 10.1109/msp.2020.2975749

[4] Zhang C, Xie Y, Bai H, Yu B, Li W, Gao Y. A survey on federated learning. Knowl-Based Syst. 2021;216:106775.

[5] Gadekallu TR, Maddikunta PKR, Boopathy P. XAI for Industry 5.0—concepts, opportunities, challenges, and future directions. IEEE Open J Commun Soc. 2025;6:2706–29.

[6] Li T, Sanjabi M, Beirami A, Smith V. Fair resource allocation in federated learning. arXiv preprint 2019. https://arxiv.org/abs/1905.10497

[7] Dinh CT, Tran NH, Nguyen MNH, Hong CS, Bao W, Zomaya AY, et al. Federated learning over wireless networks: convergence analysis and resource allocation. IEEE/ACM Trans Netw. 2021;29(1):398–409. doi: 10.1109/tnet.2020.3035770

[8] Wang C, Liang C, Yu FR, Chen Q, Tang L. Computation offloading and resource allocation in wireless cellular networks with mobile edge computing. IEEE Trans Wirel Commun. 2017;16(8):4924–38.

[9] Tran TX, Pompili D. Joint task offloading and resource allocation for multi-server mobile-edge computing networks. IEEE Trans Veh Technol. 2019;68(1):856–68. doi: 10.1109/tvt.2018.2881191

[10] Zhou X, Liu C, Zhao J. Resource allocation of federated learning for the metaverse with mobile augmented reality. IEEE Trans Wireless Commun. 2024:1. doi: 10.1109/twc.2023.3326884

[11] Zhang J, Liu Y, Qin X, Xu X, Zhang P. Adaptive resource allocation for blockchain-based federated learning in Internet of Things. IEEE Internet Things J. 2023;10(12):10621–35. doi: 10.1109/jiot.2023.3241318

[12] Hou X, Wang J, Jiang C, Meng Z, Chen J, Ren Y. Efficient federated learning for metaverse via dynamic user selection, gradient quantization and resource allocation. IEEE J Select Areas Commun. 2024;42(4):850–66. doi: 10.1109/jsac.2023.3345393

[13] Salh A, Ngah R, Audah L, Kim KS, Abdullah Q, Al-Moliki YM, et al. Energy-efficient federated learning with resource allocation for green IoT edge intelligence in B5G. IEEE Access. 2023;11:16353–67. doi: 10.1109/access.2023.3244099

[14] Wang Q, Guo S, Liu J, Pan C, Yang L. Profit maximization incentive mechanism for resource providers in mobile edge computing. IEEE Trans Serv Comput. 2022;15(1):138–49. doi: 10.1109/tsc.2019.2924002

[15] Huang X, Zhang B, Li C. Incentive mechanisms for mobile edge computing: present and future directions. IEEE Network. 2022;36(6):199–205.

[16] Wang Q, Guo S, Liu J, Pan C, Yang L. Motishare: incentive mechanisms for content providers in heterogeneous time-varying edge content market. IEEE Trans Services Comput. 2021;16(1):452–65.

[17] Li G, Cai J. An online incentive mechanism for collaborative task offloading in mobile edge computing. IEEE Trans Wireless Commun. 2020;19(1):624–36. doi: 10.1109/twc.2019.2947046

[18] Zhan Y, Zhang J, Hong Z, Wu L, Li P, Guo S. A survey of incentive mechanism design for federated learning. IEEE Trans Emerg Topics Comput. 2021;10(2):1035–44.

[19] Chen Z, Liao G, Ma Q, Chen X. Adaptive privacy budget allocation in federated learning: a multi-agent reinforcement learning approach. In: ICC 2024 -IEEE International Conference on Communications. IEEE; 2024. p. 5166–71.

[20] Tang X, Yu H. Multi-session multi-objective budget optimization for auction-based federated learning. In: 2024 International Joint Conference on Neural Networks (IJCNN). IEEE; 2024. p. 1–8.

[21] Li Z, Ding B, Yao L, Li Y, Xiao X, Zhou J. Performance-based pricing for federated learning via auction. Proc VLDB Endowm. 2024;17(6):1269–82.

[22] Sun Y, Li B, Yang K, Bi X, Zhao X. TiFLCS-MARP: client selection and model pricing for federated learning in data markets. Exp Syst Appl. 2024;245:123071. doi: 10.1016/j.eswa.2023.123071

[23] Wang X, Zheng S, Duan L. Dynamic pricing for client recruitment in federated learning. IEEE/ACM Trans Networking. 2024;32(2):1273–86. doi: 10.1109/tnet.2023.3312208

[24] Tan X, Lim WYB, Niyato D, Yu H. Reputation-aware opportunistic budget optimization for auction-based federation learning. In: 2023 International Joint Conference on Neural Networks (IJCNN). 2023. p. 1–8. 10.1109/ijcnn54540.2023.10191418

[25] Liu X, Chen H, Liu Y, Wei W, Xue H, Xia F. Multitask data collection with limited budget in edge-assisted mobile crowdsensing. IEEE Internet of Things J. 2024;11(9):16845–58.

[26] Chen L, Yang Y, Xu Q. Retail dynamic pricing strategy design considering the fluctuations in day-ahead market using integrated demand response. Int J Electric Power Energy Syst. 2021;130:106983.

[27] Wang D, Fan R, Yang P. Research on floating real-time pricing strategy for microgrid operator in local energy market considering shared energy storage leasing. Appl Energy. 2024;368:123412.

[28] Furió D, Moreno-del-Castillo J. Dynamic demand response to electricity prices: evidence from the Spanish retail market. Utilities Policy. 2024;88:101763. doi: 10.1016/j.jup.2024.101763

[29] Kim HJ, Kim MK. New customized bidirectional real-time pricing mechanism for demand response in predictive home energy management system. IEEE Internet Things J. 2024;11(14):24497–510. doi: 10.1109/jiot.2024.3381606

[30] Ibrahim M, Ekin S, Imran A. Buyers collusion in incentivized forwarding networks: a multi-agent reinforcement learning study. IEEE Trans Mach Learn Commun Netw. 2024;2:240–60.

[31] Tinoco S, Abeliuk A, del Solar JR. Impact of price inflation on algorithmic collusion through reinforcement learning agents. arXiv preprint 2025. doi: arXiv:2504.05335

[32] Lin Z, Liu Y, Xie J. Market distortions with collusion of agents. J Banking Financ. 2024;162:107151.

[33] Jang B, Park S, Lee J, Hahn SG. Three hierarchical levels of big-data market model over multiple data sources for Internet of Things. IEEE Access. 2018;6:31269–80.

[34] Lee J, Kim D, Niyato D. Market analysis of distributed learning resource management for internet of things: a game-theoretic approach. IEEE Internet Things J. 2020;7(9):8430–9. doi: 10.1109/jiot.2020.2991725

[35] Black F, Jones RW. Simplifying portfolio insurance. JPM. 1987;14(1):48–51. doi: 10.3905/jpm.1987.409131

[36] Tao M, Li X, Ota K, Dong M. Single-cell multiuser computation offloading in dynamic pricing-aided mobile edge computing. IEEE Trans Comput Soc Syst. 2024;11(2):3004–14. doi: 10.1109/tcss.2023.3308563

[37] Weida L, Hongbin D. Pricing research on spatial crowdsourcing tasks under incompletely uncertain scene information. In: 2023 IEEE International Conference on Systems, Man, and Cybernetics (SMC). 2023. p. 534–41. 10.1109/smc53992.2023.10393968

[38] Pindyck RS. Microeconomics. Pearson; 2018.

[39] Gale D. The law of supply and demand. Math Scand. 1955;3:155. doi: 10.7146/math.scand.a-10436

[40] Quinlan S. The popularity factor: a comparative investigation of demand-side behavioral personalization. Politic Stud. 2024. doi: 00323217241300991

[41] Wang Q, Chen P, Liu J, Wang Y, Guo Z. Investment-driven budget allocation and dynamic pricing strategies in edge cache network. Pervas Mobile Comput. 2025;109:102040. doi: 10.1016/j.pmcj.2025.102040

[42] Wang X, Wang L, Yu Z, Xu Z, Zhang Y, Chu W. Pricing in the open market of crowdsourced video edge caching: a newcomer perspective. In: 2022 IEEE International Performance, Computing, and Communications Conference (IPCCC). 2022. p. 263–8. 10.1109/ipccc55026.2022.9894319

[43] Liu J, Li T, Wang Q, Wang Y, Guo Z, Yu K. Unleashing collaborative potentials: multifaceted collaboration among agents in multitask internet of things networks. IEEE Internet of Things J. 2025.

[44] Ma G, Hu M, Wang X, Li H, Bian Y, Zhu K, et al. Joint partial offloading and resource allocation for vehicular federated learning tasks. IEEE Trans Intell Transport Syst. 2024;25(8):8444–59. doi: 10.1109/tits.2024.3393529

[45] Chen X, Li Z, Ni W, Wang X, Zhang S, Sun Y, et al. Toward dynamic resource allocation and client scheduling in hierarchical federated learning: a two-phase deep reinforcement learning approach. IEEE Trans Commun. 2024;72(12):7798–813. doi: 10.1109/tcomm.2024.3420733

